# Lauric Acid Is an Inhibitor of *Clostridium difficile* Growth *in Vitro* and Reduces Inflammation in a Mouse Infection Model

**DOI:** 10.3389/fmicb.2017.02635

**Published:** 2018-01-17

**Authors:** Hsiao-Ting Yang, Jenn-Wei Chen, Jagat Rathod, Yu-Zhen Jiang, Pei-Jane Tsai, Yuan-Pin Hung, Wen-Chien Ko, Daniel Paredes-Sabja, I-Hsiu Huang

**Affiliations:** ^1^Department of Microbiology and Immunology, College of Medicine, National Cheng Kung University, Tainan, Taiwan; ^2^Center of Infectious Disease and Signaling Research, National Cheng Kung University, Tainan, Taiwan; ^3^Department of Earth Sciences, National Cheng Kung University, Tainan, Taiwan; ^4^Department of Medical Laboratory Science and Biotechnology, College of Medicine, National Cheng Kung University, Tainan, Taiwan; ^5^Department of Internal Medicine, National Cheng Kung University Hospital, Tainan, Taiwan; ^6^Department of Internal Medicine, Tainan Hospital, Ministry of Health and Welfare, Tainan, Taiwan; ^7^Graduate Institute of Clinical Medicine, National Cheng Kung University Hospital, Tainan, Taiwan; ^8^Center of Infection Control, National Cheng Kung University Hospital, Tainan, Taiwan; ^9^Department of Medicine, College of Medicine, National Cheng Kung University, Tainan, Taiwan; ^10^Microbiota-Host Interactions and Clostridia Research Group, Departamento de Ciencias Biológicas, Facultad de Ciencias Biológicas, Universidad Andrés Bello, Santiago, Chile

**Keywords:** *Clostridium difficile*, medium-chain fatty acid, lauric acid, alternative therapy, natural product

## Abstract

*Clostridium difficile* is a Gram-positive, spore-forming anaerobic human gastrointestinal pathogen. *C. difficile* infection (CDI) is a major health concern worldwide, with symptoms ranging from diarrhea to pseudomembranous colitis, toxic megacolon, sepsis, and death. CDI onset and progression are mostly caused by intestinal dysbiosis and exposure to *C. difficile* spores. Current treatment strategies include antibiotics; however, antibiotic use is often associated with high recurrence rates and an increased risk of antibiotic resistance. Medium-chain fatty acids (MCFAs) have been revealed to inhibit the growth of multiple human bacterial pathogens. Components of coconut oil, which include lauric acid, have been revealed to inhibit *C. difficile* growth *in vitro*. In this study, we demonstrated that lauric acid exhibits potent antimicrobial activities against multiple toxigenic *C. difficile* isolates *in vitro*. The inhibitory effect of lauric acid is partly due to reactive oxygen species (ROS) generation and cell membrane damage. The administration of lauric acid considerably reduced biofilm formation and preformed biofilms in a dose-dependent manner. Importantly, in a mouse infection model, lauric acid pretreatment reduced CDI symptoms and proinflammatory cytokine production. Our combined results suggest that the naturally occurring MCFA lauric acid is a novel *C. difficile* inhibitor and is useful in the development of an alternative or adjunctive treatment for CDI.

## Introduction

*Clostridium difficile* is a Gram-positive, spore-forming bacillus that was first isolated from the gut of an infant and became medically important when it was found to be the leading cause of antibiotic-associated diarrhea (AAD) in hospital settings worldwide (Smits et al., 2016). It is estimated that 15–25% of AAD cases can be attributed to *C. difficile* infection (CDI) (Ananthakrishnan, 2011). Partly due to increased awareness and diagnosis, the incidence and economic burden of CDI have increased yearly, and CDI cases have been reported in every continent (Burke and Lamont, 2014; Desai et al., 2016). Prior antibiotic exposure, advanced age (more than 65 years), prior hospitalization, the presence of an underlying illness, and proton pump inhibitor use have all been identified as risk factors for CDI (Vardakas et al., 2012; McDonald et al., 2015).

The principal virulence factors of *C. difficile* are two large cytotoxins, toxin A and toxin B, which have been reported to exhibit enterotoxigenic and cytotoxic activity (Pruitt et al., 2010; Abt et al., 2016). Both toxins are capable of severely inflaming the colon and disrupting the epithelial mucosal surface. According to the current guideline for CDI treatment, prior antibiotic retreatment should be discontinued and replaced with metronidazole as first-line treatment; vancomycin is administered for extremely severe cases or relapses (Surawicz et al., 2013). Recurrence, one of the hallmarks of CDI, is due to the ability of *C. difficile* to produce stress-resistant spores and partly due to the inability of the gut flora to be restored after antibiotic treatment; recurrence can occur in 25% of patients with CDI, and the rate can increase up to 40–60% following a second recurrence (Johnson, 2009). The recently Food and Drug Administration (FDA)-approved antibiotic fidaxomycin has been identified as having similar treatment effects as those of vancomycin while having a reduced impact on the gut flora (Louie et al., 2012), although the recurrence rate is still high. Several vaccines developed by pharmaceutical companies are currently being tested in clinical trials, but no active immunization therapies have been approved by the FDA. However, bezlotoxumab, an antitoxin B monoclonal antibody, has been approved for preventing CDI recurrence (Martin and Wilcox, 2016; Villafuerte Galvez and Kelly, 2017). Alternative treatment and preventive strategies against CDI are therefore required.

Various free fatty acids (FAs), as well as their monoglyceride derivatives, have long been known to exert antimicrobial effects on numerous bacterial pathogens (Galbraith et al., 1971; Kabara et al., 1972). Short-chain fatty acids (SCFAs) such as acetic, propionic, and butyric acid have been proven to exhibit antibacterial activity against various pathogens including *Vibrio parahaemolyticus, C. perfringens, Salmonella*, and *Helicobacter pylori* (Thompson and Hinton, 1997; Namkung et al., 2011; Immanuel et al., 2012; Yonezawa et al., 2012). Of all medium-chain fatty acids (MCFAs) tested *in vitro*, lauric acid (dodecanoic acid, C12:O) and capric acid (decanoic acid, C10:O) have been demonstrated to have the most potent effect against various bacterial, fungal, and viral pathogens (Kabara et al., 1972; Bartolotta et al., 2001; Bergsson et al., 2001; Rouse et al., 2005; Huang et al., 2011). Lauric acid, in particular, has been revealed to exhibit antibacterial activity against both Gram-positive and Gram-negative pathogens such as *Staphylococcus aureus, Streptococcus mutans, S. pyogenes, Escherichia coli, H. pylori*, and many others (Kabara et al., 1972; Rouse et al., 2005). A recent study demonstrated the bactericidal effects of MCFAs (caprylic, capric, and lauric acid) combined with edible plant essential oils (carvacrol, eugenol, β-resorcylic acid, *trans*-cinnamaldehyde, thymol, and vanillin) against *E. coli* O157:H7 (Kim and Rhee, 2016). As the primary FA of coconut oil is lauric acid (45–53%), it is of great interest to utilize coconut oil as a source of lauric acid. The antimicrobial properties of lauric acid, monolaurin, and their ester derivatives may be attributed to physicochemical processes as well as their interference with various cellular processes (Dayrit, 2015). Shilling et al. (2013) reported that MCFAs can inhibit the growth of the *C. difficile* strain ATCC 9689 *in vitro*, and that lauric acid exhibits the highest potency. However, the extent of and the mechanism by which lauric acid inhibits *C. difficile* and its destructive effects on bacterial physiology and the spore outgrowth ability have not been investigated comprehensively, and importantly, the effect of lauric acid on CDI *in vivo* has not been analyzed.

In the present study, we screened multiple FAs for their ability to inhibit *C. difficile* growth *in vitro* and confirmed that lauric acid exerts the highest inhibitory effect. A comprehensive analysis demonstrated that lauric acid could inhibit biofilm formation and reduce spore outgrowth. Mechanistic studies revealed that the inhibition of *C. difficile* was partly due to the generation of intracellular reactive oxygen species (ROS) and membrane damage. In a mouse infection model, lauric acid consumption decreased CDI-induced colon inflammation and diarrhea, supporting the hypothesis that lauric acid is a potential compound for CDI treatment.

## Materials and Methods

### *C. difficile* Strains and Culturing Conditions

Eleven *C. difficile* strains were utilized in this study. Strains R20291 (ribotype 027, *tcdA*^+^, *tcdB*^+^, *tcdC*^+^, *cdtA*^-^, *ctdB*^+^) and 630 (ribotypes 012, *tcdA*^+^, *tcdB*^+^, *tcdC*^+^, *ctdA*^-^, *ctdB*^-^) used in this study are described elsewhere (McEllistrem et al., 2005; Buckley et al., 2011). The other nine isolates, which consisted of three tcdA+tcdB+ isolates (TNHP 29, 59, and 207), three tcdA-tcdB+ isolates (TNHP 79, 82, 403), and three tcdA-tcdB- isolates (1, 3, and 6), were originally isolated from a hospital in Southern Taiwan and have been described by Hung et al. (2016). All *C. difficile* strains were cultured anaerobically on brain–heart infusion (BHI) agar or in BHI broth (Thermo Fisher Scientific, Waltham, MA, United States) supplemented with 0.05% L-cysteine. Anaerobic experiments were conducted inside a Don Whitley DG250 anaerobic workstation (Don Whitley Scientific Ltd., West Yorkshire, United Kingdom).

### Fatty Acid Minimum Inhibitory Concentration (MIC), Minimum Bactericidal Concentration (MBC), and Half Maximal Inhibitory Concentration (IC_50_) Determination

Fatty acids (propionic acid, butyric acid, isobutyric acid, valeric acid, isovaleric acid, hexanoic acid, octanoic acid, capric acid, lauric acid, myristic acid, and palmitic acid) were purchased from Sigma–Aldrich (St. Louis, MO, United States). A minimum inhibitory concentration (MIC) assay was conducted according to the guidelines of the Clinical Laboratory and Standards Institute (formerly the National Committee for Clinical Laboratory Standards) for anaerobes. An overnight-grown culture was refreshed 50-fold in fresh brain–heart infusion-supplemented (BHIS) broth, incubated until the optical density at 600 nm (OD_600_) was approximately 0.35, and then diluted 8-fold in 96-well microplates. FAs predissolved in dimethyl sulfoxide (DMSO) were then added to the bacterial suspension to reach a 5% DMSO final concentration. Concentrations of FAs ranged from 0.01 to 5 mg/mL. Bacterial cells were also incubated in BHIS + 1% DMSO only as a control. Plates were incubated anaerobically at 37°C for 24 h. To determine the MBC, bacterial suspensions from each well were streaked out onto BHIS agar plates and incubated for an additional 24 h. The MBC of each FA was defined as the lowest concentration at which no visible colony was observed. To determine the inhibitory effects of different concentrations of lauric acid on the *C. difficile* strain R20291, the percentage of growth at each concentration was calculated using the following equation: inhibition (%) = [1 - (OD_600_ of growth with lauric acid/OD_600_ of growth in broth only) × 100]. The growth inhibition rate was plotted against the log of the lauric acid concentration, and the IC_50_ value was defined as the value that caused a 50% reduction in bacterial growth. At least three independent samples were analyzed for each experiment.

### Growth Curves of Various Concentrations of Lauric Acid Incubated with *C. difficile*

To determine the antibacterial activity of lauric acid on *C. difficile* growth, we employed different concentrations of lauric acid (1×, 4×, and 8× MBC) in the growth assay conducted in an anaerobic chamber. In the assay, 1% DMSO served as a treatment control. The *C. difficile* strain R20291 was cultured in BHIS broth at 37°C in the anaerobic chamber for 16 h, and the overnight cultures were then refreshed 50-fold in fresh BHIS broth until the late exponential to early stationary phase (OD_600_ = approximately 0.8). Next, bacterial suspensions were added to DMSO or lauric acid, and OD595 was determined using a Libra S2 Colorimeter (Biochrom, Cambridge, United Kingdom). At least three independent samples were analyzed for each experiment.

### Biofilm Assay

To determine the effect of lauric acid on biofilm formation, an overnight culture of the *C. difficile* strain R20291 was refreshed to the late exponential to early stationary phase (OD_600_ = approximately 0.8) in BHIS broth and then diluted 100-fold in fresh medium (BHIS + 0.1 M glucose) in 24-well polystyrene plates. Lauric acid at concentrations ranging from 0.125× to 1× MBC was added, and plates were incubated anaerobically at 37°C for 72 h. To determine the effect of lauric acid on preformed biofilms, biofilms were prepared for 24 h before treatment with lauric acid at concentrations ranging from 1× to 4× MBC for another 24 h. Moreover, 20 μg/mL vancomycin [40× the MBC of strain R20291, (Dapa et al., 2013)] and 1% DMSO were used as controls. To quantify the biofilm mass, supernatants were carefully decanted, and biofilms that formed in all wells were allowed to dry at room temperature. Two percent crystal violet (CV) was added to each well for 30 min and then removed through methanol treatment for an additional 30 min. Extracted dye contents were quantified by measuring the absorbance at 595 nm by using a Multiskan^TM^ GO Microplate Spectrophotometer (Thermo Fisher Scientific). Experiments were performed at least three times.

### Spore Preparation

Spores were prepared by plating a 1:100 dilution of overnight culture onto BHIS agar plates and then incubating the plates for 10 days at 37°C under anaerobic conditions. Spores were harvested with ice-cold sterile distilled water and purified with 50% Nicodenz (Axis Shield, Oslo, Norway) using a previously described method (Sorg and Sonenshein, 2008). Spores were purified to >99% purity as determined using phase contrast microscopy, and the number of spores per milliliter was quantified through visual enumeration using a Neubauer Chamber (Sigma–Aldrich) prior to use.

### Spore Germination and Outgrowth Assay

To monitor germination efficiency, purified spores were first heat activated by incubating them for 30 min at 60°C and were then adjusted to an OD_600_ of 1.0 in sterile water; 75-μL aliquots of spore suspensions were mixed with equal volumes of lauric acid or sterile water supplemented with 5% DMSO for 20 min at room temperature. Next, 10 mM taurocholic acid (Fisher Scientific) was added, and OD_600_ was measured at 2-min intervals (Multiskan^TM^ GO Microplate Spectrophotometer, Thermo Fisher Scientific). The ratio of the OD_600_ at time X to the OD_600_ at time zero (t0) was plotted against time. The level of dipicolinic acid (DPA) release was monitored in real-time through terbium fluorescence, as described in previous studies (Bhattacharjee et al., 2015; Francis et al., 2015). Briefly, 75 μL of purified spores previously adjusted to the OD_600_ of 1.0 were resuspended in germination buffer (10 mM Tris-Cl, 150 mM NaCl, 100 mM glycine, pH 7.5) and were then treated with equal volumes of various concentrations of lauric acid for 20 min. Next, 10 mM taurocholic acid was added, and DPA release was monitored using a FlexStation^®^ 3 Multi-Mode Microplate Reader (Molecular Devices, Sunnyvale, CA, United States) at an excitation wavelength of 270 nm and an emission wavelength of 545 nm. Spores boiled at 100°C for 30 min served as a positive control for total DPA release. Statistical analysis was performed using GraphPad Prism version 6.0. Three independent experiments were conducted. DPA release was calculated using the following equation:

$$\begin{array}{c} \mathrm{DPA}\mathrm{release}(\%)=\frac{{\mathrm{RFU}}_{\mathrm{sample}}}{\mathrm{Average}\mathrm{of}{\mathrm{RFU}}_{\mathrm{boiled}\;\mathrm{spores}}}\times 100\% \end{array}$$

To measure spore outgrowth, from the aforementioned germination assay, a 100-μL aliquot of spores from each reaction was serially diluted (10^-1^–10^-7^) with sterile phosphate-buffered saline (PBS) and then spread onto BHIS agar plates supplemented with 0.l% sodium taurocholate (TA) and incubated anaerobically at 37°C overnight. Spore outgrowth was calculated using the following equation:

$$\begin{array}{c} \mathrm{Spore}\mathrm{outgrowth}(\%)=\frac{\mathrm{CFU}{\mathrm{count}}_{\mathrm{lauric}\;\mathrm{acid}\;\mathrm{treated}\;\mathrm{sample}}}{\mathrm{Average}\mathrm{of}\mathrm{CFU}{\mathrm{count}}_{\mathrm{DMSO}\;\mathrm{control}}}\times 100\% \end{array}$$

### Cytoplasmic Material Leakage Measurement

An overnight culture of *C. difficile* R20291 was diluted at 1:50 in fresh BHIS broth and then grown to an OD_600_ of approximately 0.8. Bacterial cells were then treated with various concentrations of lauric acid (1×–8× MBC) anaerobically at 37°C. Moreover, 1% DMSO in PBS served as the negative control, and 100 μg/mL nisin (Sigma–Aldrich) served as the positive control. At various time points, supernatants were collected, and the absorbances at 260 nm were recorded using a BioPhotometer UV/Visible Spectrophotometer (Eppendorf, Hamburger, Germany). Three independent experiments were performed.

### Live/Dead Bacterial Viability Measurement

To measure cell viability, an overnight culture of *C. difficile* R20291 was diluted at 1:50 in fresh BHIS broth and then grown to an OD_600_ of approximately 0.8. Bacterial cells were then treated with lauric acid at 0.25× MBC or 1% DMSO in PBS for 20 min. Bacterial pellets were collected and resuspended in sterile PBS. Suspensions were then mixed with the LIVE/DEAD BacLight staining reagent mixture (Molecular Probes, Invitrogen) according to the manufacturer’s instructions. Samples were visualized under a FluoView^TM^ FV1000 confocal microscope (Olympus), and fluorescence was detected at an excitation wavelength of 488 nm and an emission wavelength of 500 nm (SYTO9) and 635 nm (propidium iodide). Cell viability is expressed as the ratio of SYTO-9-stained cells to the total number of cells. At least three independent trials were performed for each experiment.

### Ultrathin-Section Transmission Electron Microscopy

Transmission electron microscopy (TEM) was employed to visualize the cells damaged by lauric acid treatment. *C. difficile* R20291 cells grown to the exponential phase were concentrated through centrifugation and treated with 0.25× MBC of lauric acid anaerobically for 15 min. Samples were embedded in Embed-812 (Electron Microscopy Sciences) and cut with an EM UC6 ultramicrotome (Leica, Wetzlar, Germany). Sections with a thickness of 90 nm were placed on copper grids (Electron Microscopy Sciences) and then stained with 2% uranyl acetate and lead citrate. Ultrathin sections were examined under a JEM-1400 transmission electron microscope (JEOL) with 120 kV acceleration and a 4k × 4k CCD Camera System Model 895 (Gatan, Inc.). The results are representative of three independent experiments.

### Fluorescent Dye-Based Detection of ROS

Reactive oxygen species was measured using the carboxy derivative of fluorescein, CM-H2DCFDA (Life Technologies), according to the protocol provided by the manufacturer with the following modification: briefly, an overnight-grown *C. difficile* culture was refreshed to OD_600_ of approximately 0.8 in BHIS broth. Moreover, 198 μL of the bacterial suspension was incubated with 2 μL of stock CM-H2DCFDA anaerobically at 37°C for 30 min. Cells were then treated with 1× MBC of lauric acid for 10 min, and fluorescence was then measured using the FlexStation^®^ 3 Multi-Mode Microplate Reader (Molecular Devices) at an excitation wavelength of 488 nm and an emission wavelength of 535 nm. The following controls were included: bacterial suspensions in BHIS broth containing 1% DMSO as the negative control; bacterial suspensions in BHIS broth containing 0.0035% hydrogen peroxide solution (H_2_O_2_; Sigma–Aldrich) and 10 mM tert-butyl hydroperoxide solution (TBHP; Sigma–Aldrich) as the positive control. The results are representative of three independent experiments.

### Bacterial RNA Extraction and Real-Time Quantitative Reverse Transcription Polymerase Chain Reaction

An overnight culture of *C. difficile* R20291 was refreshed in Tryptone Yeast or BHIS broth and was grown anaerobically at 37°C until OD_600_ was approximately 0.8. Bacterial cells were treated with various concentrations of lauric acid or 1% DMSO (control group) for 30 min anaerobically at 37°C. Cells were harvested through centrifugation, and total RNA was isolated using the RNAprotect Bacteria Reagent (QIAGEN, Venlo, Netherlands) in accordance with the manufacturer’s instructions. Genomic DNA was removed using RQ1 RNase-free DNase (Promega). RNA was reverse transcribed into complementary DNA (cDNA) by using SuperScript^TM^ II Reverse Transcriptase (Invitrogen) and random primers (Thermo Fisher Scientific) according to the manufacturer’s instructions. The relative transcriptional level of putative ROS-related genes between the control group and the lauric acid treatment group was measured through real-time quantitative reverse transcription polymerase chain reaction (qRT-PCR) using the 2x qPCRBIO SyGreen Mix Hi-Rox (PCR Biosystems) and gene specific primers (**Table 1**), according to the manufacturer’s instructions. A StepOnePlus^TM^ Real-Time PCR System (Applied Biosystems) was used. The data were analyzed using the 2^-ΔΔ^*^C^*^t^ method, with normalization to the reference gene 16s and the stated reference condition. Samples were analyzed in at least three independent trials. Statistical analyses were performed using GraphPad Prism 6.0.

**Table 1:** Sequences of oligonucleotide primers used in this study.

Name	Sequence (5′–3′)	Species	Reference
16s-F	GAT TTA CTT CGG TAA AGA GCG G	*C. difficile*	This study
16s-R	CCT TAC CAA CTA GCT AAT CAG ACG	*C. difficile*	This study
TcdA-F	AAA GCT TTC GCT TTA GGC AGT G	*C. difficile*	This study
TcdA-R	CTC TAT GGC TGG GTT AAG GTG TTG	*C. difficile*	This study
TcdB-F	GAT CAC TTC TTT TCA GCA CCA TCA	*C. difficile*	This study
TcdB-R	AGC TTC TTA AAC CTG GTG TCC ATC	*C. difficile*	This study
CD1529-F	TGT CTT TGG TTC TGG TTG GG	*C. difficile*	This study
CD1529-R	ACT TAC AGG GCT ATC CTG ATT TG	*C. difficile*	This study
CD0757-F	GAC TTG TGG AAA CCT TGT AGG A	*C. difficile*	This study
CD0757-R	TGC TGC ATC TGT TGT ATT AGG A	*C. difficile*	This study
CD1716-F	CTG ACC CTG ACT TAG TTG CTA TAA A	*C. difficile*	This study
CD1716-R	ATA TGT CGC ACG TAC AAC TCC	*C. difficile*	This study
CD1465-F	GCT ATG CAA TAC TTG TCC CAA AG	*C. difficile*	This study
CD1465-R	GCT AAG CTC TTC TGC TGC TAT	*C. difficile*	This study
mβ-actin-F	ACT GCC GCA TCC TCC TCC TC	Mouse	Hung et al., 2015
mβ-actin-R	TGC CAC AGG ATT CCA TAC CC	Mouse	Hung et al., 2015
mTNF-α-F	CAT CTT CTC AAA ATT CGA GTG ACA A	Mouse	Hung et al., 2015
mTNF-α-R	TGG GAG TAG ACA AGG TAC AAC CC	Mouse	Hung et al., 2015
mIL-6-F	AGG ATA CCA CTC CCA ACA GAC	Mouse	Hung et al., 2015
mIL-6-R	GTG CAT CAT CGT TGT TCA TAC	Mouse	Hung et al., 2015
mIL-1β-F	GCA ACT GTT CCT GAA CTC AAC T	Mouse	Hung et al., 2015
mIL-1β-R	ATC TTT TGG GGT CCG TCA AT	Mouse	Hung et al., 2015
mMIP-2-F	TGT CAA TGC CTG AAG ACC CTG CC	Mouse	Hung et al., 2015
mMIP-2-R	AAC TTT TTG ACC GCC CTT GAG AGT GG	Mouse	Hung et al., 2015
mMCP-1-F	CCC ACT CAC CTG CTG CTA CT	Mouse	Hung et al., 2015
mMCP-1-R	TCT GGA CCC ATT CCT TCT TG	Mouse	Hung et al., 2015

### An Animal Model of CDI

Specific-pathogen-free 8-week-old male C57BL/6 mice were housed in the Laboratory Animal Center of (NCKU). All mice were maintained and handled according to the guidelines of the Institutional Animal Care and Use Committee (IACUC) of NCKU. All animal studies were performed following the protocol approved by the IACUC of NCKU (approval NCKU-IACUC-102-149) and the Biosafety and Radiation Safety Management Division of NCKU. The animal model of CDI was established as previously described (Chen et al., 2008; Liu et al., 2017; Pizarro-Guajardo et al., 2017). Five animals were administered 12 mg/kg (low dose) and 24 mg/kg (high dose) of lauric acid dissolved in PBS orogastrically once per day for 7 days prior to infection with *C. difficile* and once more 1 day following infection. To condition the animals for CDI, mice were fed drinking water containing an antibiotic mixture, which included 0.4 mg/mL vancomycin, 0.215 mg/mL metronidazole, 0.4 mg/mL kanamycin, 0.035 mg/mL gentamicin, and 850 U/mL colistin, for a total of 5 days before the challenge. All antibiotics were purchased from Sigma–Aldrich. On the day before the challenge, mice were fed the antibiotic mixture without vancomycin and metronidazole, which were excluded to avoid disrupting *C. difficile* colonization. Esomeprazole dissolved in PBS was given to all mice through oral gavage 12 h prior to infection (18.55 mg/kg) and immediately before infection (4.82 mg/kg). On the day of infection, 1 × 10^6^ CFU of *C. difficile* R20291 spores were administered through oral gavage, and 4 mg/kg of clindamycin was injected intraperitoneally. Two days after infection, all animals were euthanized through CO_2_ asphyxiation. The severity of diarrhea was scored in accordance with mice stool consistency, as follows: (0) well-formed pellets; (1) semiformed stools that did not adhere to the anus; (2) semiformed stools that adhered to the anus; and (3) liquid stools. Organs and gastrointestinal lavage (GAL) fluids were extracted for downstream analysis. The entire animal experiment was performed for a total of three independent sets, and representative results were obtained.

### Fecal Colony-Forming Unit Determination

Fecal samples (premixed in PBS) were collected from the animals, immediately heat treated at 65°C for 20 min, and then serially diluted onto BHI agar containing 0.1% TA. Plates were incubated anaerobically at 37°C for 48 h, and colonies were counted for CFU determination.

### Mice Colon RNA Extraction and Real-Time Quantitative Reverse Transcription Polymerase Chain Reaction

Colon samples were extracted using RNeasy^®^ Plus Mini kits (QIAGEN). RNA yield and quality were examined using a NanoDrop Spectrophotometer (Thermo Fisher Scientific). Reverse transcription was performed with SuperScript^TM^ II Reverse Transcriptase (Invitrogen, Waltham, MA, United States). The expression levels of proinflammatory cytokines and chemokines were measured through qRT-PCR using RealQ Plus 2X Master Mix Green (Ampliqon, Denmark), with β-actin as the reference gene in each reaction (**Table 1**). The data were analyzed using the ΔΔ*C*t method and expressed as the fold change in the transcription level under the test condition compared with the average for the indicated control and were then normalized to the reference gene β-actin. Statistical analyses were performed using GraphPad Prism 6.0.

### Cytokine and Chemokine Measurement

The concentrations of GAL cytokines and chemokines were measured using a DuoSet^®^ enzyme-linked immunosorbent assay (ELISA) development system (R&D Systems, Minneapolis, MN, United States) according to the manufacturer’s instructions. Absorbance was measured at 450 nm using an iMark^TM^ microplate reader (Bio-Rad, Hercules, CA, United States). Samples were measured in triplicate, and statistical analyses were performed using GraphPad Prism version 6.0.

### Statistics

All data are expressed as the mean ± standard deviations of at least three independent experiments. Statistical comparisons among the groups were made using Student’s *t*-test. Multiple intergroup comparisons were made using one-way analysis of variance (ANOVA), followed by a *post hoc* Tukey’s test with GraphPad Prism version 6.0. Statistical significance was set at *P* < 0.05.

## Results

### Antibacterial Activity of Free FAs against *C. difficile* R20291

To identify the free FAs with potent *C. difficile* inhibitory effects, FAs C3–C16 were coincubated with log-phase-grown *C. difficile* cells, and their MBCs were determined (**Table 2**). For the test strain R20291, butyric acid (MBC = 50 mg/mL) showed the least inhibitory effects of all FAs tested, whereas lauric acid (MBC = 0.3125 mg/mL) showed the most potent effects. The length of the carbon chain did not appear to be an influencing factor contributing to the antibacterial activity of the FAs, although remarkably MCFAs appeared to exhibit lower MBCs. Specifically, the MBCs of both capric acid (C10) (1.25 mg/mL) and lauric acid (C12) (0.3125 mg/mL) were the second lowest and the lowest of all FAs tested. Moreover, the resulting pH variation in BHIS medium did not appear to contribute to the antibacterial activity of the various FAs (**Table 2**). As the MBC of lauric acid was significantly lower (fourfold, *P* < 0.0001) than that of capric acid, lauric acid was chosen as a potential candidate for subsequent experiments.

**Table 2:** Antibacterial activity of fatty acids against *C. difficile* strain R20291.

	Number of carbon backbone	General name	MIC (mg/ml)	MBC (mg/ml)	pH (in BHIS)
SCFAs	C3	Propionic acid	1.25	5	3.91
	C4	Butyric acid	25	50	7.11
	C4	Isobutyric acid	1.25	5	4.97
	C5	Valeric acid	1.25	2.5	5.78
	C5	Isovaleric acid	2.5	2.5	5.76
MCFAs	C6	Hexanoic acid	1.25	2.5	5.94
	C8	Octanoic acid	2.5	5	5.51
	C10	Capric acid	0.63	1.25	6.81
	C12	Lauric acid	0.31	0.31	6.91
LCFAs	C14	Myristic acid	>10	>10	6.51
	C16	Palmitic acid	10	10	6.77

*Clostridium difficile strain R20291 was exposed to various dilutions of different fatty acids for 24 h, and MBC was determined as the concentration of fatty acids at which no visible growth was observed. Results are expressed as average of at least three independent replicates. SCFA, short-chain fatty acids; MCFA, medium-chain fatty acids; LCFA, long-chain fatty acids.*

**FIGURE 1:**
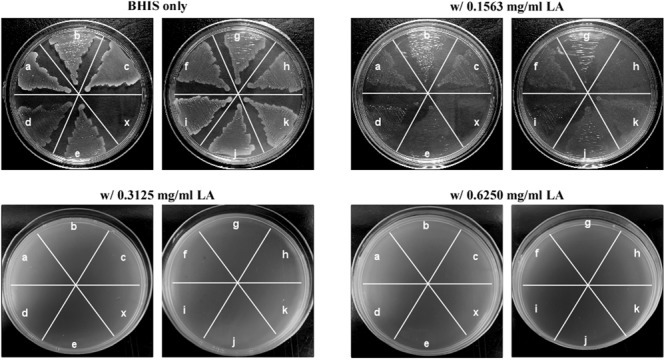
Lauric acid is a potent inhibitor of multiple *Clostridium difficile* clinical isolates. Growth inhibition of multiple *C. difficile* isolates on BHI agar plates containing lauric acid at 0.5× (0.1563 mg/mL), 1× (0.3125 mg/mL), and 2× (0.625 mg/mL) MBC. a: TNHP 207; b: TNHP 59; c: TNHP 20; d: 630; e: R20291; f: TNHP 403; g: TNHP 82; h: TNHP 79; i: TNHP1; j: TNHP 3; k: TNHP 6; x: negative control. Results are representative of at least three independent experiments.

**FIGURE 2:**
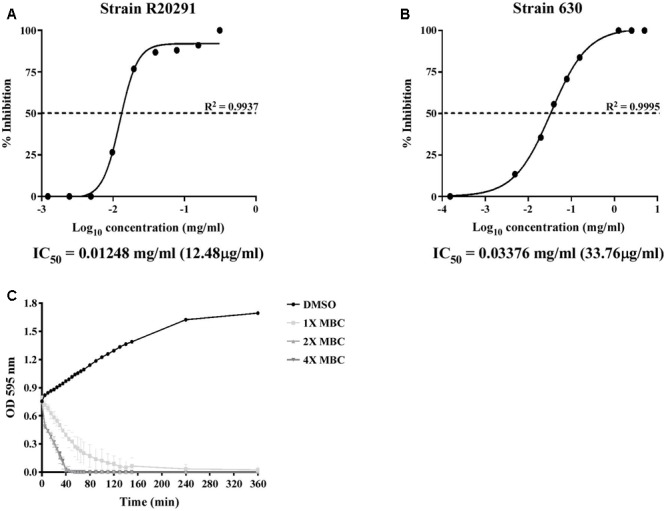
IC_50_ determination and time-dependent antibacterial kinetic curve of lauric acid against *C. difficile*. **(A)** *C. difficile* strains R20291 and 630 were treated with various concentrations of lauric acid for 24 h, and the OD at 600 nm was then measured. Growth inhibition was normalized to the 5% DMSO control group. **(B)** Log-phase grown *C. difficile* R20291 cells were incubated with various concentrations of lauric acid in BHI broth for up to 6 h. **(C)** *C. difficile* R20291 cells were incubated with 1×, 2×, and 4× MBC of lauric acid, and growth was monitored over time by measuring OD595. DMSO was included as a control. Results are expressed as mean of triplicate samples at least three independent experiments.

To further confirm the inhibitory effect of lauric acid on *C. difficile* growth, 10 additional clinical isolates of different toxinotypes were subjected to the same experiment (**Table 3** and **Figure 1**) (Hung et al., 2016). The MICs of all 11 tested isolates ranged from 0.08 to 0.16 mg/mL; however, the MBCs were all 0.31 mg/mL. As the MBCs were all the same, we assumed the inhibitory effect of lauric acid on *C. difficile* is likely not strain-dependent; hence, all subsequent experiments were performed using the laboratory strains 630 and R20291. To gain further insight into the inhibitory effect of lauric acid on *C. difficile*, the IC_50_ was determined. As depicted in **Figures 2A,B**, the IC_50_ of lauric acid against *C. difficile* strains R20291 and 630 was 12.48 and 33.67 μg/mL, respectively. The antibacterial activity of lauric acid against strain R20291 was further evaluated in liquid nutrient broth containing different concentrations of lauric acid (**Figure 2C**). When lauric acid was applied at 2× and 4× MBC, cell lysis appeared to occur immediately, as evidenced by the drop in OD. In short, lauric acid was revealed to display an inhibitory effect on multiple strains of *C. difficile*, and the inhibition mechanism is likely bactericidal.

**Table 3:** Inhibition of *C. difficile* strains by lauric acid.

*C. difficile* strain	Toxin genotype	MIC (mg/ml)	MBC (mg/ml)	Ribotype
R20291	*tcdA*+*tcdB*+ (laboratory strains)	0.08	0.31	RT 027
630		0.08	0.31	RT 012
TNHP 20	*tcdA*+*tcdB*+ (clinical isolate strains)	0.16	0.31	RT 002
TNHP 59		0.08	0.31	RT 002
TNHP 207		0.16	0.31	RT 106
TNHP 79	*tcdA*-*tcdB*+ (clinical isolate strains)	0.08	0.31	RT 017
TNHP 82		0.08	0.31	RT 017
TNHP 403		0.16	0.31	RT 017
TNHP 1	*tcdA-tcdB-* (isolated from asymptomatic adults)	0.08	0.31	ND
TNHP 3		0.16	0.31	ND
TNHP 6		0.16	0.31	ND

*Clostridium difficile isolates containing two laboratory strains and nine local isolates were exposed to various concentration of lauric acid for 24 h. MBC was determined as described previously. Results are expressed as average of at least three independent replicates. ND – not determined.*

### Effects of Lauric Acid Treatment on Biofilm Formation and Stability

**FIGURE 3:**
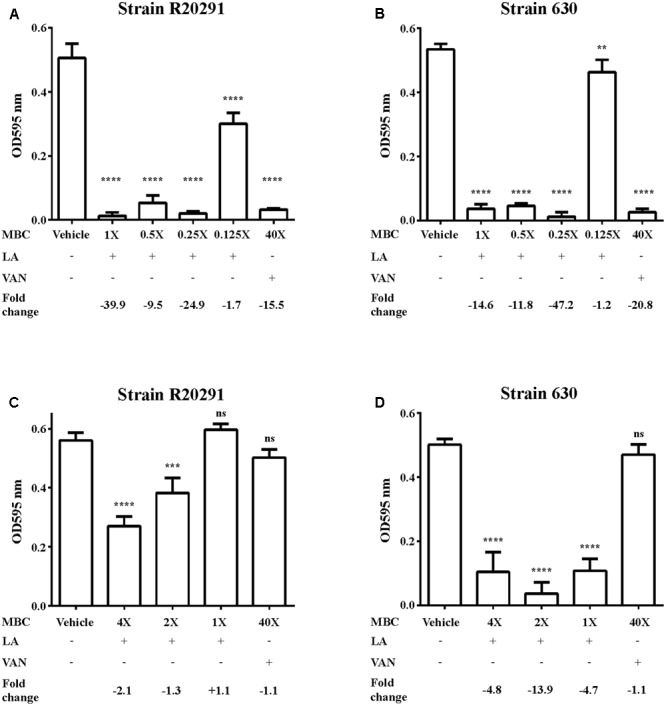
Inhibition of *C. difficile* biofilm by lauric acid. Various concentrations of lauric acid were added to strains R20291 **(A)** and 630 **(B)** grown in multi-well plates, and adherent biofilms were quantified by CV staining. The effect of lauric acid on preformed biofilms was measured by incubating various concentrations of lauric acid with a 24-h old biofilm formed by strains R20291 **(C)** and 630 **(D)** for an additional 24 h. The disruption of the preformed biofilm was quantified through CV staining. Vehicle: 1% DMSO only. LA: lauric acid. VAN: 20 μg/mL vancomycin (40× MBC). Lauric acid MBC: 4× = 1.25 mg/mL, 2× = 0.63 mg/mL, 1× = 0.31 mg/mL, 0.5× = 0.16 mg/mL, 0.25× = 0.08 mg/mL, 0.125× = 0.04 mg/mL. Results are the mean of triplicate samples, and one-way ANOVA was performed to assess significance. (ns, no significance; ^∗∗^*P* < 0.01, ^∗∗∗^*P* < 0.001, ^∗∗∗∗^*P* < 0.0001).

To further understand the effect of lauric acid on *C. difficile*, we determined whether lauric acid treatment affects biofilm formation and stability. In this study, we measured the effect of lauric acid treatment on biofilm formation by R20291 and 630 strains, as these two strains were reported to exhibit different biofilm forming abilities (Dapa et al., 2013). Clinical *C. difficile* strains are known to form robust biofilms *in vitro*, and these biofilm-dwelling cells are more resistant to antibiotics and perhaps to even host defenses than planktonic cells are (Dapa and Unnikrishnan, 2013; Dapa et al., 2013; Crowther et al., 2014). A previous study reported that vancomycin applied at 20 μg/mL (100× MIC) can significantly reduce the survival of *C. difficile* biofilm cells (Dapa and Unnikrishnan, 2013). For the strain R20291, vancomycin applied at 100× MIC could reduce biofilm formation by 15.5-fold compared with the DMSO control (*P* < 0.0001). However, 0.25× MBC of lauric acid could significantly reduce biofilm formation by 24.9-fold compared with the control (*P* < 0.0001) (**Figure 3A**). Similarly, for the strain 630, although vancomycin treatment led to a 20.8-fold reduction in biofilm formation, 0.25× MBC of lauric acid led to a 47.2-fold reduction (**Figure 3B**). To determine whether lauric acid treatment also disrupts established biofilms, a static culture of *C. difficile* was grown in multi-well plates for 24 h before lauric acid addition. Interestingly, although vancomycin applied at 100× MIC did not reduce the biofilm mass, lauric acid at least 2× MBC and 1× MBC could reduce the mass of biofilms formed by strains R20291 and 630, respectively (**Figures 3C,D**). Reduction of the mass of biofilms formed by the strain R20291 required a higher concentration of lauric acid than that from strain 630.

### Effect of Lauric Acid Treatment on Spore Germination and Outgrowth

**FIGURE 4:**
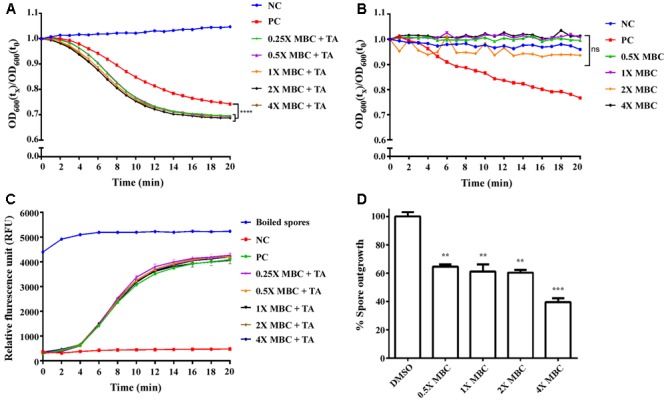
Effect of lauric acid on *C. difficile* spore germination. Heat-activated spores from the *C. difficile* strain R20291 were incubated with 10 mM sodium taurocholate plus 5% DMSO (positive control, PC), 5% DMSO only (negative control, NC), or various concentrations of lauric acid plus 10 mM sodium taurocholate and 5% DMSO. Germination was monitored by measuring absorbance at 600 nm **(A,B)** or DPA release **(C)**. **(D)** Spore outgrowth was assayed by incubating spores in the presence of various concentrations of lauric acid for 20 min, and CFU/mL was determined by plating aliquots of spores onto BHIS agar containing TA. Results are the mean of triplicate samples, and one-way ANOVA was performed to assess significance. (ns, no significance; ^∗∗^*P* < 0.01, ^∗∗∗^*P* < 0.001, ^∗∗∗∗^P < 0.0001).

In addition to biofilm formation, the spore-forming ability of *C. difficile* contributes to its transmission (Deakin et al., 2012). Spores are known to be resistant to multiple environmental stresses, including cold, heat, desiccation, antiseptics, and antibacterial products (Rodriguez-Palacios and Lejeune, 2011; Deng et al., 2015; Edwards et al., 2016), and they are therefore a critical component of the pathogenesis of CID. One of the key germinating signals for *C. difficile* spores is the presence of TA. In the presence of TA, spores will undergo core hydration, and this process can be visualized microscopically; previously dormant phase-dark spores will become phase-bright due to core hydration and the eventual degradation of the cortex peptidoglycan. To measure the effect of lauric acid on spore germination, purified *C. difficile* strain R20291 spores were treated with taurocholic acid and various concentrations of lauric acid. As revealed in **Figure 4A**, in the presence of TA, the OD_600_ of the spore suspension decreased significantly within 20 min of exposure (PC group, *P* < 0.0001), whereas the DMSO control remained phase-dark (NC group). Interestingly, when various concentrations of lauric acid were added along with TA, the decrease in OD_600_ was faster over time than that in the PC group, suggesting that core degradation occurred at a faster rate (*P* < 0.0001 for all concentrations compared with the PC group). We also performed the same assay in the absence of TA, and no significant decrease in OD_600_ was observed for all concentrations of lauric acid tested throughout the 20 min of observation, suggesting that the effect was TA-dependent (**Figure 4B**). During spore germination, DPA release can be measured and is often used as a sign of spore germination. To further investigate the role of lauric acid in spore germination, we measured DPA released for 20 min (**Figure 4C**). As expected, DPA released from boiled spores was detected by its high fluorescence signals, which were higher than those of non-treated spores. Similarly, in the presence of TA, an increase in the fluorescent signal was detected, suggesting that DPA release was initiated at approximately 4 min and continued to increase until the end of the experiment. However, the addition of lauric acid, regardless of concentration, did not significantly alter DPA release over the course of the experiment (*P* > 0.9999). Furthermore, the addition of lauric acid alone did not induce any DPA release (data not shown). Finally, the viability of spores in the presence of lauric acid was measured (**Figure 4D**). Compared with spores exposed to TA only, lauric acid treatment in the presence of TA considerably decreased the rate of spore outgrowth in a dose-dependent manner. Spore outgrowth decreased to 64.5, 61, 60.4, and 39.6% of typical growth when subjected to lauric acid concentrations of 0.5× MBC, 1× MBC, 2× MBC, and 4× MBC, respectively (**Figure 4D**). These combined results demonstrate that lauric acid treatment can inhibit *C. difficile* biofilm formation and stability and can affect spore outgrowth.

### Lauric Acid Inhibited *C. difficile* by Inducing ROS Generation and Cell Membrane Damage

**FIGURE 5:**
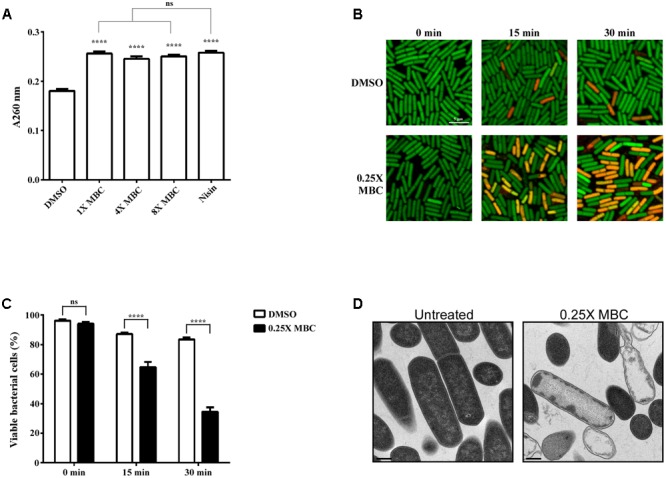
Lauric acid induces bacterial cell membrane damage. To measure the damaging effect of lauric acid on *C. difficile* cell membrane, the vegetative cells of the strain R20291 were treated with various concentrations of lauric acid for up to 30 min, and cellular material leakage was quantified by measuring absorbance at 260 nm **(A)**. Nisin served as positive control. **(B)** Membrane permeability was measured by incubating cells with sublethal concentrations of lauric acid (0.25× MBC) for 15 and 30 min. Cells were then stained with SYTO9 (green) and propidium iodide (red) and imaged with confocal microscopy at 1,000× magnification. Scale bar = 5 μM. **(C)** Bacterial viability was quantified by counting the number of green fluorescent and red fluorescent cells from six images. **(D)** TEM analysis of vegetative cells treated with 0.25× MBC for 15 min compared with untreated control. Images were taken at 10,000× magnification, and scale bars = 0.5 μM. Results are the mean of three independent experiments. One-way ANOVA and two-way ANOVA was performed to assess significance for **(A)**, and **(C)**, respectively. ns, no significance; ^∗∗∗∗^*P* ≤ 0.0001.

To determine whether the inhibitory effect of lauric acid on *C. difficile* growth is due to the disruption of cell membrane integrity, *C. difficile* cells were treated with lauric acid, and the extracellular presence of released nucleic acid was measured (**Figure 5A**). In this assay, we included nisin, a broad-spectrum polycyclic antibacterial peptide produced by *Lactococcus lactis*, as a positive control, as it is known to attack bacterial cell membranes; this leads to cytoplasmic content release and cell lysis eventually (Ruhr and Sahl, 1985; Nobmann et al., 2010). As expected, in the presence of the antibacterial peptide nisin, a significant quantity of nucleic acids was detected in the culture supernatant at 30 min after treatment. When cells were treated with various concentrations of lauric acid, a considerably higher quantity of nucleic acid materials was also detected compared with the negative control, indicating that the addition of lauric acid compromised cell membrane integrity. The viability of *C. difficile* cells treated with lauric acid was further assessed using LIVE/DEAD staining and was visualized using confocal microscopy (**Figure 5B**). As lauric acid induced the rapid lysis of *C. difficile* cells, as indicated in **Figure 2C**, to elucidate the effects of lauric acid on *C. difficile* cells, these cells were treated with sublethal concentrations of lauric acid (0.25× MBC). When cell viability was quantified based on the percentage of cells that stained positive for propidium iodide, treatment with lauric acid at 0.25× MBC decreased viability to approximately 65% by 15 min after treatment (**Figure 5C**). By 30 min after treatment, the viability decreased to approximately 35%. By contrast, the viability of the DMSO-treated control remained relatively high (15 min: 87%, 30 min: 84%). Ultrathin-section TEM analysis revealed that 20 min of lauric acid treatment (0.25× MBC) was sufficient to induce substantial cell death, as indicated by abnormal cell morphology and cytoplasmic content leakage. These findings are similar to those of Shilling et al. (2013) (**Figure 5D** and Supplementary Figure 1).

**FIGURE 6:**
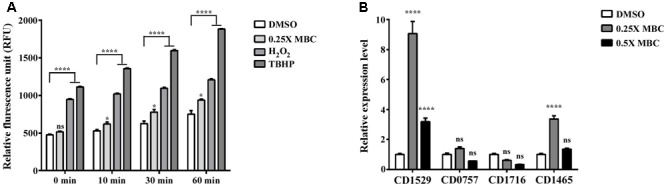
Effect of lauric acid on intracellular ROS production and ROS-related genes in *C. difficile*. **(A)** *C. difficile* cells were treated with lauric acid for up to 60 min, and intracellular ROS was determined by staining with the general ROS indicator CM-H2DCFDA. H2O2 and TBHP served as ROS induction control. 1% DMSO – negative control. **(B)** The effect of lauric acid on ROS-related genes in *C. difficile*. *C. difficile* cells were treated with lauric acid or 1% DMSO for 30 min, and gene expression was then measured using real-time polymerase chain reaction. CDR20291_1529 (putative superoxide dismutase), CDR20291_0757 (putative superoxide reductase), CDR20291_1716 (putative peroxidase), and CDR20291_1465 (putative catalase). All data are presented as mean ± standard deviations, and statistical comparisons among groups were made using one-away ANOVA (^∗^*p* ≤ 0.05, ^∗∗∗∗^*p* ≤ 0.0001). ns, not significant. All data are representative of at least three independent experiments.

To determine whether lauric acid also induces ROS generation, vegetative *C. difficile* R20291 cells were treated with a sublethal concentration of lauric acid (0.25× and 0.5× MBC) for up to 60 min, and intracellular ROS levels were measured (see section “Materials and Methods”). As depicted in **Figure 6A**, treatment with the antiseptic hydrogen peroxide and tert-butyl hydroperoxide (TBHP) generated a considerable level of ROS in a time-dependent manner. Interestingly, lauric acid treatment also generated a substantial level of ROS over the course of the experiment. As no molecular or biochemical studies have been performed in *C. difficile* on ROS-associated genes, from the genomic annotation, we selected four potential antioxidant defense-associated genes. As depicted in **Figure 6B**, in the presence of 0.25× MBC of lauric acid, the expression levels of genes encoding for a putative superoxide dismutase (locus tag CDR20291_1529) and a putative catalase (locus tag CDR20291_1465) were upregulated (9.1- and 3.4-0 fold, respectively, compared with untreated cells) (**Figure 6B**). However, the gene expression levels of CDR20291_C0757, which encodes for a putative superoxide reductase, and CDR20291_1716, which encodes for a putative peroxidase, were not significantly altered in the presence of either 0.25× or 0.5× MBC of lauric acid. Nevertheless, the results of these experiments suggested that the bactericidal activity of lauric acid was partly due to the induction of membrane damage and ROS generation, which resulted in cell lysis.

### Lauric Acid Pretreatment Decreased *C. difficile*-Induced Inflammation in a Mouse Infection Model

**FIGURE 7:**
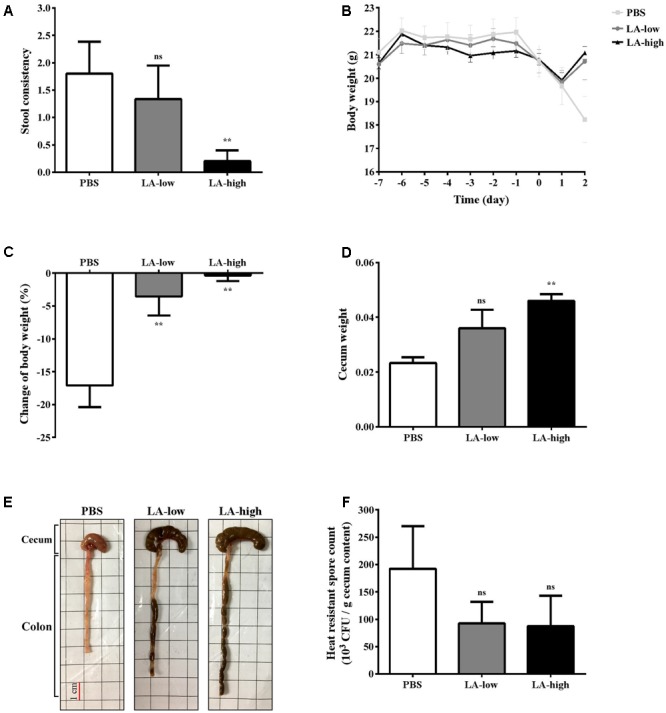
Lauric acid treatment protects mice from *C. difficile* infection. Various groups of mice receiving lauric acid or PBS only were treated with an antibiotic cocktail and then challenged by *C. difficile* for 2 days. Stool consistency **(A)**, body weight over time **(B)**, body weight change **(C)**, cecum weight **(D)**, gross views of colon and cecum **(E)**, and heat-resistant fecal spore count 2 days post infection **(F)** were assessed. PBS: mice receiving PBS pre-treatment only; LA-low: mice receiving 12 mg/kg lauric acid; LA-high: mice receiving 24 mg/kg lauric acid. All data are presented as mean ± standard deviations, and statistical comparisons among groups were made using one-way ANOVA (*n* = 5, ^∗∗^*P* ≤ 0.01). ns, not significant. All data are representative of at least three independent experiments.

Finally, to determine whether lauric acid affects CDI *in vivo*, C57BL/6 mice were administered lauric acid orogastrically for 1 week prior to infection with purified *C. difficile* R20291 spores (Supplementary Figure 2). Mice administered only PBS prior to infection displayed symptoms of CDI, including a lack of well-formed feces due to diarrhea and a considerable decrease in body and cecum weight (**Figures 7A–D**). Gross views of the colon and cecum indicated severe colitis (**Figure 7E**). By contrast, both groups of mice that were administered either 12 mg/kg (LA-low) or 24 mg/kg (LA-high) of lauric acid displayed healthier colon and cecum morphology (**Figure 7E**), body weight recovery after infection (**Figure 7B**), a substantially lower body weight decrease (**Figure 7C**), and a higher cecum weight (**Figure 7D**) than the PBS control group. The protective effects of lauric acid appeared to be dose-dependent, as mice belonging to the LA-high group appeared to exhibit less severe symptoms of CDI compared with mice in the LA-low group. No differences were observed in the number of heat-resistant spores recovered from fecal samples (**Figure 7F**).

**FIGURE 8:**
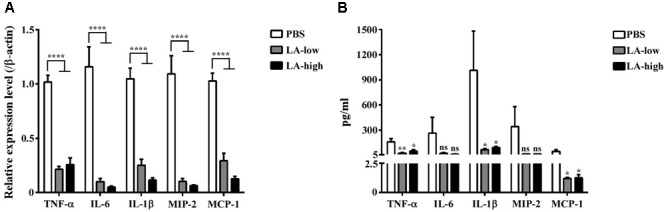
Decreased level of proinflammatory cytokines in *C. difficile*-infected mice receiving lauric acid. The level of various proinflammatory cytokines and chemokines in colon tissues **(A)** and GAL **(B)** from mice belonging to the PBS treatment group, LA-low (12 mg/kg) group, and LA-high (24 mg/kg) group was measured using real-time polymerase chain reactions and ELISA, respectively. All data are presented as mean ± standard deviations, and statistical comparisons among groups were made using Student’s *t*-test (^∗^*P* ≤ 0.05, ^∗∗^*P* ≤ 0.01, ^∗∗∗∗^*P* ≤ 0.0001). ns, not significant. All data are representative of at least three independent experiments.

The expression levels of genes encoding for proinflammatory cytokines such as tumor necrosis factor α (TNF-α), interleukin 6 (IL-6), interleukin 1β (IL-1βb), macrophage inflammatory protein 2 (MIP-2), and monocyte chemoattractant protein 1 (MCP-1) were considerably decreased in the colon homogenates of the LA-low and LA-high groups compared with those in the PBS group (**Figure 8A**). In addition to gene expression, proinflammatory cytokines and chemokines from the GAL fluid of all three groups were analyzed. Levels of TNF-α, IL-1β, and MCP-1 were significantly decreased in the GAL lavage fluid of both lauric acid treatment groups compared with that in the PBS control group (*P* = 0.0254, *P* = 0.036, and *P* = 0.0285, respectively, compared with the PBS control group) (**Figure 8B**). No statistically significant differences were observed in the levels of IL-6 and MIP-2 detected, although the trend was similar to that observed above. Collectively, these results demonstrated that the administration of lauric acid could decrease the severity of *C. difficile*-induced inflammation *in vivo*.

## Discussion

The main three drug resistance strategies of *C. difficile* are drug inactivation, target modification, and efflux pump, which have led to the emergence of hypervirulent drug-resistant strains (Harnvoravongchai et al., 2017). Noticeably, in recent years, the ability of *C. difficile* to tolerate multiple commonly prescribed antibiotics, its production of potent cytotoxins (toxin A, toxin B, and binary toxin CdtAB), and its high recurrence rate have resulted in CDIs becoming a healthcare concern worldwide (Martin et al., 2016). The current guideline for CDI treatment has focused on discontinuing previous antibiotic usage, and switching the treatment to metronidazole and vancomycin (Ananthakrishnan, 2011). However, similar to various other bacterial pathogens, *C. difficile* can develop antibiotic resistance; therefore, alternative treatment or prevention strategies are required. The development of new antibiotics, such as fidaxomicin, and monoclonal antibodies, such as bezlotoxumab, has provided clinicians with additional treatment options (Miller, 2010; Wilcox et al., 2017), but the prescription costs of these new medications remain high; thus, these drugs might not be readily accessible to economically disadvantaged patients. Furthermore, most new antibiotics are derivatives of existing ones and therefore share a similar mechanism of action and risk of drug resistance. The development of novel antibacterial products that are less likely to result in drug resistance in bacteria is therefore necessary. In the current study, we evaluated the inhibitory effects of various FAs on the growth of the *C. difficile* strain R20291. In our study, MCFAs, in general, were more effective in inhibiting *C. difficile* growth than SCFAs and LCFAs, with lauric acid exhibiting the lowest MBC. In contrast to the reported effect of SCFAs on bacterial pathogens, we revealed that SCFAs did not display considerable antibacterial activity against *C. difficile*. *C. difficile*, similar to many other members of the *Clostridium* genus, is known to produce various SCFAs such as butyric acid; therefore, *C. difficile* might have developed resistance mechanisms against these FAs (May et al., 1994; Ferreyra et al., 2014; Pettit et al., 2014).

Lauric acid is the major component of coconut oil, an edible oil extracted from the meat of coconuts. Lauric acid accounts for 45–53% of the overall FA composition of coconut oil; therefore, coconut oil is a dietary supplement that can modulate serum cholesterol levels (Katan et al., 1994; German and Dillard, 2004). When lauric acid is ingested, it is released from its triglyceride form and can either enter the liver through a portal vein or can be reformed into new triglycerides and enter the lymphatic system (Dayrit, 2015). In the serum, lauric acid is known to oxidize rapidly; therefore, only a small amount enters the liver. Once inside the liver, lauric acid is metabolized into acetyl-CoA for energy production, and some reaction products can also be transformed into ketone bodies, which also aid in energy production. The perception that lauric acid has beneficial effects is derived from studies that have indicated that lauric acid consumption increased serum high density lipoprotein (HDL), which is known to decrease the risk of coronary heart diseases (de Roos et al., 2001; Ekanayaka et al., 2013; Eyres et al., 2016). In addition to their ability to modulate cholesterol levels, the antimicrobial activity of MCFAs has been established for many years. Kabara et al. (1972) reported that compared with the other MCFAs screened (C6–C18), lauric acid showed the most potent effects *in vitro* against Gram-positive bacteria (Lynch et al., 1983; Carpo et al., 2007). Furthermore, the 1-monoglyceride form of lauric acid, monolaurin, exhibited an even higher potency, although its antibacterial range was reduced. Lauricidin^TM^, which is composed of pure monolaurin, was patented as a nutritional supplement. Numerous studies have since been published on the antimicrobial activities of MCFAs against both Gram-negative and Gram-positive pathogens, with MBC values ranging from 0.068 to 0.375 mg/mL (Ruzin and Novick, 2000; Bergsson et al., 2001; Hinton and Ingram, 2006; Kitahara et al., 2006; Carpo et al., 2007; Nakatsuji et al., 2009; Fischer et al., 2012; Theinsathid et al., 2012). Shilling et al. (2013) reported that coconut-derived lauric acid, capric acid, and caprylic acid could inhibit the growth of *C. difficile in vitro*, whereas predigested virgin coconut oil exhibited a similar effect, although to a lesser degree. Shilling et al. (2013) also reported that lauric acid at 250 μM (MIC) could reduce bacterial growth by 90%. Although these previous studies have demonstrated the inhibitory effects of lauric acid in its pure form or as a derivative of lipolyzed virgin coconut oil, a research gap exits; none of these studies have revealed lauric acid’s mode of action, its effect on CD physiology, and its effects on CDIs. In our study, we extended the antibacterial activity of lauric acid to multiple clinical isolates that included both toxigenic and non-toxigenic strains, and we revealed that the MBC ranged from 0.312 to 0.625 mg/mL, which supports the results of previous studies. We noted that the IC_50_ values for strain 630 and R20291 were considerably different, even though the MBC values for both strains were the same. Several possible reasons can be provided for this discrepancy. We used a more conservative approach for determining MBC; that is, we used the concentration at which no any growth was observed on agar plates. In addition, it has been reported that the MIC values for two bacterial strains were the same, whereas their IC_50_ values were considerably different (Barbour et al., 2016). In addition to the inhibitory effect observed on vegetative cells, lauric acid could inhibit biofilm formation. At 0.25× MBC, lauric acid was equally as effective at reducing biofilm formation as vancomycin applied at 20 μg/mL. This effect was probably due to the inhibition of all cell growth. More interestingly, lauric acid disrupted preformed biofilms, and this biofilm-damaging effect had not been reported in other studies. Dapa et al. (2013) reported that R20291 *in vitro* forms biofilms with higher mass than strain 630 does, which corroborates our observation that the preformed biofilm of the strain 630 was significantly disrupted by 1× MBC of lauric acid, in contrast with the biofilm of the strain R20291, which required 2× MBC of lauric acid for a disruptive effect. Future studies should investigate the effect of lauric acid on biofilm reduction by comparing the presence of live or dead cells in the retained biofilm. At present, the exact mechanism underlying biofilm removal by lauric acid is unclear. It is possible that the mild detergent effect of lauric acid not only damages cell membranes, but also bacterial adhesins that contribute to biofilm formation. Importantly, the biofilm removal effect of lauric acid supports its potential use as an antibacterial agent.

Previous studies have indicated that MCFAs, including lauric acid, can inhibit the outgrowth of *Bacillus* and *Clostridium* spores, although *C. difficile* was not included in these studies (Ababouch et al., 1992; Shearer et al., 2000). In our study, we also observed that lauric acid treatment was effective at reducing spore outgrowth. Spore germination is a dynamic process that is initiated by the hydration of the spore cortex, followed by the release of DPA, which can be monitored by changes in OD and by measuring the DPA level in the supernatant. In the present study, the rate of spore germination was increased in the presence of lauric acid, but no differences were observed in the rate of DPA release. We hypothesize that lauric acid accelerates the rate of germination in the presence of the germinant TA, and that this hastens the killing of the eventually germinated vegetative cells. Studies are currently underway to address this possibility. Additional studies are required to determine whether lauric acid or other MCFAs can disrupt bacterial spore coats to enhance spore germination.

In the present study, we observed a significant increase in membrane permeability and the release of cytoplasmic materials, consistent with the membrane-damaging effect of lauric acid reported in previous studies (Nobmann et al., 2010). Currently, it is unclear how lauric acid penetrates the cell wall to reach membrane sites. We reported almost equal nucleic acid material release rates among nisin (positive control) and all lauric acid treatment groups (1×–8× MBC). Furthermore, lauric acid significantly induced ROS generation and significantly increased the expression of genes potentially associated with oxidative damage defense. The lower expression level of these genes in cells treated with a high concentration of lauric acid than in cells treated with a lower concentration of lauric acid might be due to the rapid toxicity of lauric acid, which suppressed bacterial metabolism. Recently, Kint et al. (2017) reported that the alternative sigma factor σ^B^ is involved in protection against ROS. Interestingly, although no significant differences were observed in the expression level of CDR20291_0757 and CDR_1716 in this study, their homologs in the strain 630 were differentially regulated by σ^B^. ROS regulation may be different between the two strains, or that ROS generation induced by lauric acid in our study might induce other genes. Future studies should conduct a transcriptomic analysis to increase our understanding of the extent to which lauric acid treatment affects gene expression in *C. difficile*.

In our *in vivo* experiments, we observed that daily lauric acid intake significantly reduced the severity of diarrhea and intestinal inflammation associated with CDI. It is still unclear whether the direct killing effect of lauric acid on *C. difficile* observed *in vitro* was involved in the reduction in the inflammation observed *in vivo*. Additional animal studies should focus on increasing the sample size, a longer postinfection observation, and determining the luminal lauric acid concentration during infection. If the concentration of luminal lauric acid reaches a similar level as the MIC determined in this study, then the reduction in inflammation might be due to the direct killing of *C. difficile* in the gut. It is also possible that the luminal lauric acid concentration did not reach the MIC, which suggests that lauric acid acted upon the host to reduce inflammation. In this study, the observation that the number of fecal spores between lauric acid-treated and control groups was similar indicated an indirect effect of lauric acid *in vivo*. It has been reported that MCFAs, including lauric acid, are partial PPAR-α and PPAR-γ agonists, which are known to exert anti-inflammatory effects (Kliewer et al., 1997; Clark, 2002; Croasdell et al., 2015). However, more experiments are required to clarify these outstanding questions. Collectively, the results of this study indicate that lauric acid exhibits potent antibacterial activity against *C. difficile*, and lauric acid prophylaxis may substantially decrease the level of inflammation induced by infection with *C. difficile in vivo*. The beneficial effect of lauric acid as a food supplement or as an adjunct therapy for CDI should be considered.

## Author Contributions

I-HH, H-TY, JR, and J-WC designed the experiments. H-TY, Y-ZJ, and J-WC carried out the experiments. I-HH, H-TY, JR, J-WC, P-JT, YP-H, DP-S, and W-CK analyzed the data. H-TY, J-WC, and I-HH prepared the manuscript.

## Conflict of Interest Statement

The authors declare that the research was conducted in the absence of any commercial or financial relationships that could be construed as a potential conflict of interest. The reviewer CS and handling Editor declared their shared affiliation.
