# Supplementary material for: Lauric Acid Is an Inhibitor of Clostridium difficile Growth in Vitro and Reduces Inflammation in a Mouse Infection Model
Source: Front Microbiol. 2018 Jan 17;8:2635. doi: 10.3389/fmicb.2017.02635 (PMC5776096; doi:10.3389/fmicb.2017.02635)

Supplemental Figure 1. Ultra-thin section transmission electron microscopy analysis of C. difficile treated with lauric acid. Images were taken at 3,000X magnification and scale bars indicate 2μM.

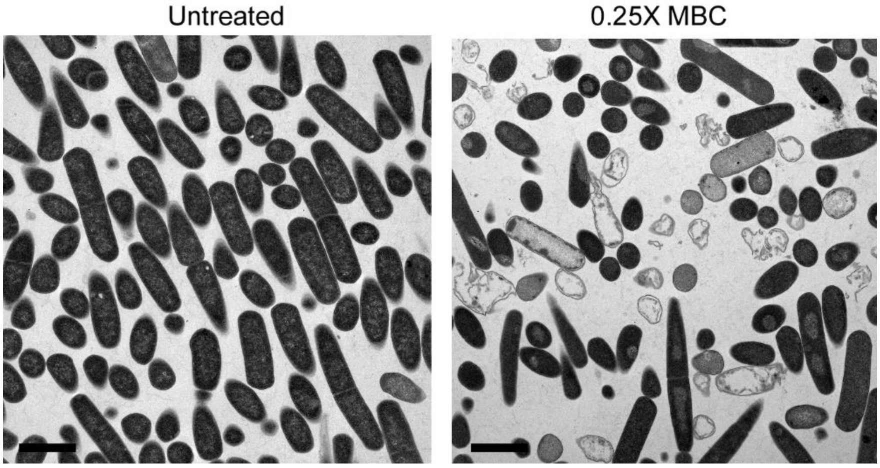

Supplemental Figure 2. Experimental design of CDI mouse model. PPI: proton pump inhibitor.

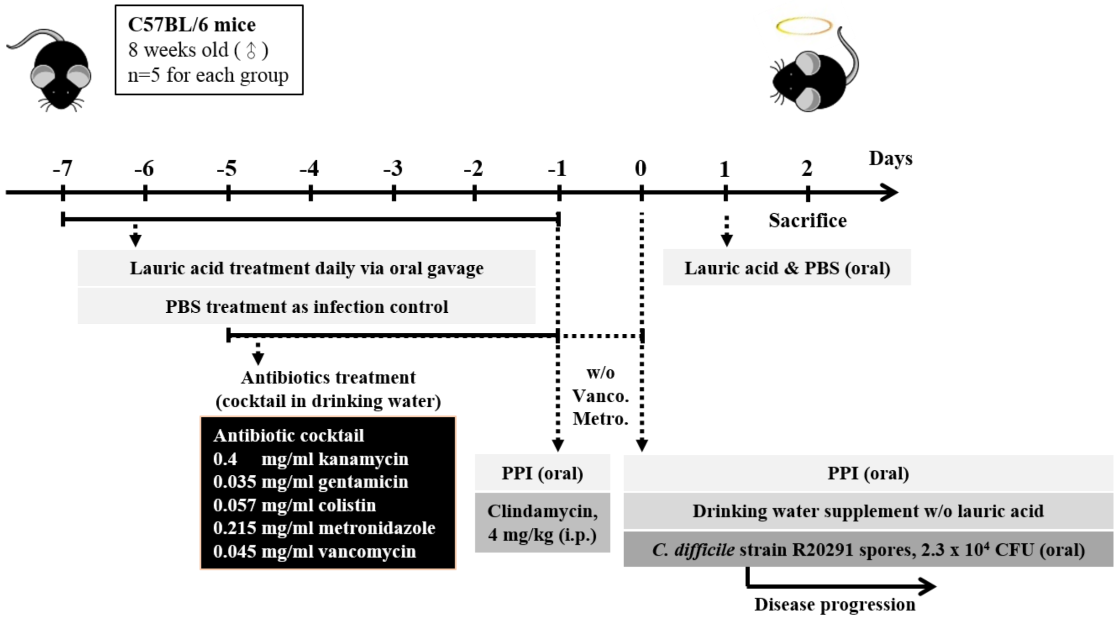

Supplement: Supplementary file 1 [file Image_1.PDF]
